# Wnt Signaling Inhibitors as Therapeutic Approach in Ischemic Heart Disease

**DOI:** 10.3390/molecules29245958

**Published:** 2024-12-17

**Authors:** Barbora Boťanská Svetláková, Viktória Pecníková Líšková, Miroslav Barančík

**Affiliations:** Centre of Experimental Medicine, Slovak Academy of Sciences, 841 04 Bratislava, Slovakia; barbora.svetlakova@savba.sk (B.B.S.); viktoria.pecnikova@savba.sk (V.P.L.)

**Keywords:** Wnt signaling, β-catenin, myocardial ischemia, Wnt inhibitors, fibrosis, pathological remodeling

## Abstract

Wnt (wingless-type MMTV integration site family) signaling is an evolutionary conserved system highly active during embryogenesis, but in adult hearts has low activities under normal conditions. It is essential for a variety of physiological processes including stem cell regeneration, proliferation, migration, cell polarity, and morphogenesis, thereby ensuring homeostasis and regeneration of cardiac tissue. Its dysregulation and excessive activation during pathological conditions leads to morphological and functional changes in the heart resulting in impaired myocardial regeneration under pathological conditions such as myocardial infarction, heart failure, and coronary artery disease. Several groups of Wnt inhibitors have demonstrated the ability to modulate the Wnt pathway and thereby significantly reduce fibrosis and improve cardiac function after myocardial ischemia. Their inhibitory effect can be realized at multiple levels, which include the inhibition of Wnt ligands, the inhibition of Frizzled receptors, the stabilization of the β-catenin destruction complex, and the disruption of nuclear β-catenin interactions. In this review, we overview the function of Wnt signaling in responses of cardiac cells to pathological conditions, especially ischemic heart disease, with an emphasis on the use of inhibitors of this signaling as a therapeutic approach. Finally, we summarize the current knowledge about the potential of the targeting of Wnt signaling in therapeutic applications.

## 1. Introduction

The maintenance of normal physiological functions of the cell is under the constant control of the systems involved in the regulation and maintenance of cellular homeostasis. The dysregulation of these systems leads to the disruption of the normal functions of the cell and to the development of pathological conditions. A significant factor, the dysregulation of which is associated with the development of pathological conditions, is signaling, which involves Wnt proteins. Dysregulation and sustained activation of Wnt signaling in the myocardium plays a key role in the pathogenesis of a variety of cardiac pathological conditions such as myocardial infarction, heart failure, cardiac hypertrophy, valvular disease, coronary artery disease, and cardiac arrhythmias [1,2]. The function of Wnt signaling during these pathological conditions is tightly connected with other regulatory processes, playing an important role in stress conditions such as redox signaling, apoptosis, and autophagy [3,4,5]. In many cases, in the therapy of pathological conditions, the application of substances that suppress the dysregulation and undesirable activation of systems involved in the maintenance of normal cellular homeostasis has an important effect. In the case of conditions with an impaired Wnt signaling function, the use of substances that are antagonists of Wnt-mediated signaling and that affect the modulation of processes that are induced by Wnt signaling seems very promising. 

In this review article, we address the role of the dysregulation of Wnt signaling in the myocardium under pathological conditions of ischemic heart disease. We overview the function of Wnt signaling with an emphasis on the use of inhibitors of this signaling as a therapeutic approach which can alter the prognosis of myocardial fibrosis and pathological tissue remodeling after myocardial ischemic injury. Finally, we summarize the current knowledge about the potential of targeting Wnt signaling in therapeutic applications. 

## 2. Wnt Signaling

The Wnt (wingless-type MMTV integration site family) signaling pathway is a highly conserved system that is strongly active during embryogenesis but is also involved in tissue regeneration and adult tissue homeostasis regulation [6,7]. The Wnt signaling pathway consists of Wnt ligand proteins, 10 different types of the Frizzled family of transmembrane receptors (Frizzled receptors, Fzd), and other signal transduction components such as scattered (Disheveled, Dvl) proteins. Currently, 19 different Wnt ligand proteins have been identified. They are polypeptides of approximately 350 residues produced by different cell types [8]. 

The Wnt/Fzd receptor complex generates diverse signals in target cells, some predominantly triggering the canonical pathway (Wnt1, Wnt2, Wnt3, Wnt3a, Wnt8a, Wnt8b, Wnt10a, and Wnt10b) and others primarily activating the noncanonical pathway (Wnt4, Wnt5a, Wnt5b, Wnt6, Wnt7a, Wnt7b, and Wnt11) [9,10,11,12]. But there is evidence that some ligands (e.g., Wnt3a, Wnt5a, and Wnt9b) function in both the canonical and noncanonical Wnt pathways [8]. Wnt signaling includes canonical and noncanonical pathways. The canonical pathway is β-catenin dependent and is known as the Wnt/β-catenin signaling pathway. In a resting state, β-catenin remains phosphorylated via a multiprotein complex called the “destruction complex”, which consists of adenomatous polyposis coli protein, axin, casein kinase 1, and glycogen synthase kinase 3β (GSK3β). Phosphorylated β-catenin is prepared for poly-ubiquitination and subsequent proteasomal degradation. The binding of Wnt ligands, which primarily activate the canonical Wnt/β-catenin pathway to G-coupled protein receptors of the Fzd family and their coreceptors LRP5/6, facilitates the recruitment of the Disheveled (Dvl) protein and axin to prevent β-catenin proteasomal degradation [9]. β-catenin can enter the nucleus, where as a second messenger it interacts with TCF/LEF transcription factors and regulates the transcription of target genes [13].

Noncanonical signaling encompasses β-catenin-independent responses elicited by Wnt ligands such as the planar cell polarity (PCP) pathway controlling cell orientation and cytoskeletal function, and Ca^2+^ pathway controlling the calcium release from the endoplasmic reticulum. The PCP pathway involves the activation of small G proteins Rac and Rho by binding the Wnt ligand to either Fzd, a receptor tyrosine kinase-like orphan receptor (ROR), or a related receptor tyrosine kinase (RYK), followed by the phosphorylation and activation of Jun-N-terminal kinase (JNK) and the subsequent transcription of Wnt target genes [14]. The Wnt/Ca^2+^ pathway involves the activation of heterotrimeric G proteins and phospholipase C (PLC), leading to phosphatidylinositol 4,5-bisphosphate cleavage into inositol 1,4,5-triphosphate (IP3) and 1,2-diacylglycerol (DAG). IP3 activates calcium channels on the endoplasmic reticulum (ER), leading to an increase in Ca^2+^ concentration. This triggers the activation of enzymes with known relevance for cardiac (patho)physiology, including calmodulin-dependent protein kinase II (CaMKII), protein kinase-C (PKC), and calcineurin, and triggers the nuclear factor of activated T cells (NFAT) to initiate the transcription of genes associated with Ca^2+^-related signaling [15]. 

## 3. Wnt Signaling in Myocardium

Both canonical and noncanonical Wnt pathways play an important role during embryonic heart development and contribute to the maintenance of adult cardiac homeostasis [16]. However, in adult hearts under normal conditions, the activities of Wnt signaling are low and reactivation occurs under pathological states or repair processes. This dysregulation of Wnt signaling plays an important role in pathological conditions such as myocardial infarction, heart failure, cardiac hypertrophy, valvular disease, coronary artery disease, and cardiac arrhythmias [1,2,17].

Ischemic heart disease (IHD), also referred as coronary heart disease (CHD) or coronary artery disease (CAD), is the leading cause of death worldwide. The term describes heart problems caused by the narrowing of the coronary vasculature, which supplies blood to the heart muscle, and includes several conditions such as sudden cardiac death, stable/unstable angina, and myocardial infarction (MI) [18]. The main causes of IHD are atherosclerosis, atherosclerotic plaque rupture, and thrombosis in the coronary arteries. As the heart does not have sufficient endogenous regenerative capacity to recover from injury, prolonged ischemia causes cardiomyocyte death, leading to a wound-healing response in the affected areas of the heart. This can ultimately lead to irreversible myocardial damage, remodeling of the remaining undamaged myocardium, and even heart failure [19]. Myocardial infarction occurs due to a reduction in coronary artery blood flow, leading to necrotic death of the cardiac muscle [20]. Acute MI remains the leading cause of cardiovascular events such as chronic heart failure and cardiac arrest. Studies have shown that the Wnt signaling pathway, particularly the canonical Wnt/β-catenin pathway, is activated following myocardial infarction [21]. This activation plays a role in orchestrating the acute inflammatory response, a critical first phase in the healing process post-MI [22].

Wnt signaling can play in myocardial responses to pathological situations both a positive and negative role. In the early phases, it serves as a potential promoter of healing and repairing of the heart after myocardial infarction, but later Wnt signaling, particularly Wnt/β-catenin signaling activation, is associated with cardiac remodeling post-injury, myocardial fibrosis development, and apoptosis, and thus negatively affects the prognosis of myocardial infarction [23,24,25,26]. In adult heart tissues, the activation of the Wnt/β-catenin pathway has been associated with fibrotic responses and pathological remodeling, suggesting its involvement in heart disease progression [27]. Several studies have indicated that the promotion of fibrosis plays an important role in the crosstalk between β-catenin and transforming growth factor-β1 (TGF-β) [28,29]. Additionally, angiogenesis in the injured myocardium may be an important biological process in myocardial repair. The newly formed vessels in the infarction area contribute to the transport of oxygen and metabolic substances in new tissues and promote tissue healing. The appearance of β-catenin in the cytoplasm of neovascular endothelial cells within 1 week after infarction was consistent with the formation time of new blood vessels around the infarcted area, indicating that it was involved in the formation of new blood vessels [30]. It has also been documented that overexpression of β-catenin enhanced the expression of VEGF and significantly increased the capillary density post-MI, which then promoted angiogenesis and tissue healing [31].

Myocardial fibrotic responses and pathological remodeling are tightly associated with abnormalities in cardiac function and the occurrence of arrhythmias. This is linked to myocardial electrical instability due to abnormal topology and the function of connexin 43 (Cx43). Cx43 is a component of gap junctions, which are intercellular channels that ensure coordinated electrical excitation and the synchronic contraction of ventricular cardiomyocytes with each heartbeat. Activation of the Wnt/β-catenin/TCF pathway in neonatal rat cardiomyocytes was confirmed to increase the expression of Cx43 and promote the colocalization of Cx43 and β-catenin in the cell membrane, enhancing intercellular coupling, which in turn negatively regulated the transcriptional activity of β-catenin [32,33]. Decreased Cx43 expression, Cx43-containing gap junction remodeling, and conduction abnormalities were observed in mouse cardiac tissue after β-catenin knockout [34]. These data suggested that β-catenin modulated arrhythmias by interacting with Cx43. Additionally, the β-catenin/cadherin complex can strengthen the connections between cells. Disruption of the β-catenin/cadherin complex leading to gap instability is one of the causes of arrhythmias [35].

## 4. Inhibitors of Wnt Signaling

**Figure 1:**
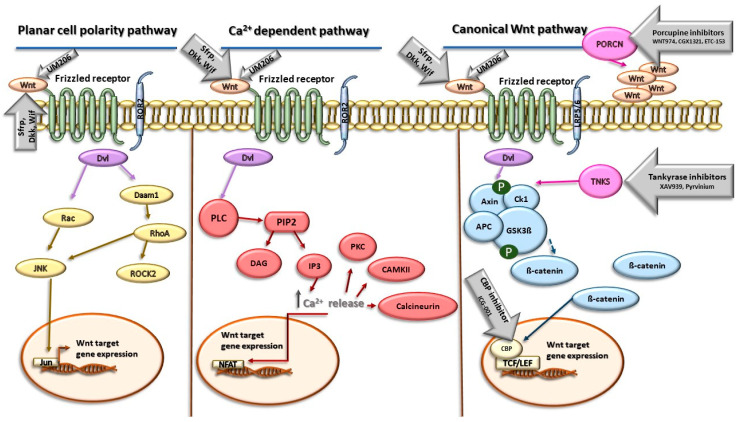
Wnt signaling pathways and the targets of their inhibitors. Abbreviations: Wnt—Wnt protein; Sfrp—secreted Frizzled-related protein; Dkk—Dickkopf family of secreted proteins; Wif—Wnt inhibitory factor; ROR2—receptor tyrosine kinase-like orphan receptor 2; PORCN—porcupine; Dvl—Disheveled; Rac—Rac protein kinase; JNK—c-Jun N-terminal kinase; Jun—Jun Proto-Oncogene; AP-1 —Transcription Factor Subunit; Daam1—Disheveled-associated activator of morphogenesis 1; RhoA—Ras homolog family member A; ROCK2—Rho-associated coiled-coil–containing protein kinase 2; PLC—phospholipase C; PIP2—phosphatidylinositol 4,5-bisphosphate; DAG—1,2-diacylglycerol; IP3—inositol 1,4,5-triphosphate; PKC—protein kinase-C; CAMKII—calmodulin-dependent protein kinase II; NFAT—nuclear factor of activated T cells; TNKS—tankyrase; Ck1—casein kinase 1; APC—adenomatous polyposis coli protein; GSK3 β—glycogen synthase kinase 3β; CBP—CREB binding protein; TCF/LEF—TCF/LEF transcription factors. Yellow color represents components of Planar cell polarity pathway, red color represents components of Ca^2+^-dependent Wnt signaling, blue color represents components of canonical Wnt signaling pathway, and gray arrows represent inhibitors of Wnt signaling and their site of action. More details are provided in the text.

Several approaches are used for targeting Wnt signaling, including small-molecule inhibitors, proteins, and peptide inhibitors. The inhibition of Wnt signaling can be realized at several levels, which involves the inhibition of Wnt ligands through the direct binding of antagonists to Wnt proteins, the inhibition of receptor phosphorylation such as transmembrane Fzd receptors, the inhibition of Dvl, the stabilization of the β-catenin destruction complex, the inhibition of β-catenin nuclear localization, and the disruption of nuclear β-catenin/TCF interactions (Figure 1).

Several groups of natural antagonists of Wnt signaling are currently known to modulate its downstream signaling. Of these, the secreted Frizzled-related protein (Sfrp), the Dickkopf (Dkk) family of secreted proteins, and Wnt inhibitory factor 1 (Wif1) have been intensively studied. The secreted Frizzled-related protein (Sfrp) represents the largest group of secreted Wnt inhibitors. This family comprises five members (Sfrp 1–5) that have a striking homology to transmembrane Fzd receptors. The structure of Sfrps contains three units as follows: an amino-terminal signal peptide consisting of 20–30 amino acids, a coiled cysteine-rich domain (CRD) and a carboxyl terminus netrin-like domain (NTR). The CRD is now usually referred to as the Frizzled domain. The domain spans approximately 120 amino acids near the N-terminus of the protein and contains 10 conserved cysteines and some additional conserved residues. Through the CRD, Sfrps can bind to the Fzd receptor, thereby competitively inhibiting the binding of Wnts to Fzd. The NTR consists of six cysteine residues, several conserved hydrophobic residues, and secondary structures, and it can extracellularly bind to Wnt, thus antagonizing Wnt signaling [36,37]. The Dickkopf (Dkk) class of proteins represents another group of Wnt antagonists. In humans, there are four Dkk proteins (Dkk1–4) and all contain two cysteine-rich domains (CRDs), designated CRD1 and CRD2, with each containing five disulfide bonds. Through these domains, Dkk binds to co-receptor LRP5/6 and antagonizes primarily the canonical Wnt signaling, acting in a competitive manner with Wnt or in a way in which Dkk sequesters LRP5/6 from the plasma membrane [38,39]. Dickkopf2 has been shown to activate LRP6/APC and stimulate angiogenic sprouting in ECs post-MI. However, the role of Dickkopf1 in regulating angiogenesis has been diminished [40]. Wif1 is a secreted 379-amino acid protein consisting of a signal peptide for extracellular secretion, the Wif domain, five EGF repeats, and a hydrophilic C-terminus. Wif1 inhibits Wnt signaling activity through direct binding to Wnt proteins via the Wif domain [36].

WNT974/LGK974, CGX1321, and ETC-153 represent another group of effective modulators of Wnt signaling [41,42,43]. The target structure of these molecules acts as membrane-bound O-acyltransferase Porcupine (PORCN), an enzyme which plays an important role in the palmitoylation of Wnt proteins. The inhibition of PORCN suppresses the secretion of Wnt ligands involved in both canonical and noncanonical Wnt pathways. 

Pyrvinium, an FDA-approved anthelmintic compound, has been identified as a Wnt pathway inhibitor that acts through the stabilization of the β-catenin destruction complex. The effects of Pyrvinium involve the inhibition of axin degradation and allosteric activation of casein kinase 1α [44,45]. Treatment with Pyrvinium in mice resulted in observable angiogenesis and Ki67 + cardiac cell regeneration in the peri- and remote myocardium post-MI, which mitigated adverse cardiac remodeling [44]. The stabilization of axin also plays a role in effects of XAV939, a small-molecular inhibitor of Wnt signaling acting through the inhibition of tankyrases (TNKS) [46]. The inhibition of these poly (ADP-ribose) polymerases results in the stabilization of axin and the promotion of β-catenin degradation. 

Another group of Wnt antagonists represents small molecules that specifically block the interaction between β-catenin and its coactivator, the CREB-binding protein (CBP). These effects are associated with an inhibition of β-catenin-mediated gene transcription. To this group of inhibitors belongs ICG-001, which specifically inhibits the interaction between β-catenin and CBP in the classical Wnt signaling pathway. Its effects are associated with the promotion of the differentiation of epicardial progenitor cells and the facilitation of myocardial regeneration and an improvement of cardiac function in rats post-MI [47]. 

UM206 is a peptide with the amino acid sequence Ac-CNKTSEGMDGCEL-NH_2_ derived from a region of high homology between multiple Wnt molecules. This peptide exhibits high homology with Wnt-3a/5a and acts as an antagonist of the Wnt signaling pathway by inhibiting the transduction of the Frizzled protein. UM206 can reduce the infarct area, increase capillary density, decrease fibroblastic cells in the infarcted heart after MI, and ultimately suppress the development of heart failure [48].

## 5. Wnt Inhibitors and Ischemic Heart Disease

**Figure 2:**
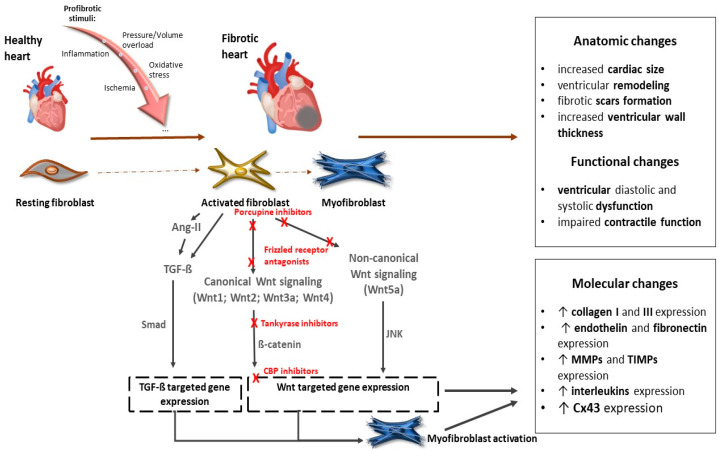
The role of the Wnt signaling pathway in the development of cardiac fibrosis and potential targets of their inhibitors. Abbreviations: Wnt—Wnt protein; Ang-II—angiotensin II; TGF-ß—transforming growth factor ß; Smad—Smad proteins; JNK—c-Jun N-terminal kinase; CBP—CREB-binding protein; MMPs—matrix metalloproteinases; TIMPs— tissue inhibitors of matrix metalloproteinases; Cx43—connexin 43. Red Xs represent sites for potential inhibition of Wnt signaling. More details are provided in the text.

Several Wnt inhibitors have shown a capacity to modulate the Wnt pathway and significantly enhance cardiac outcomes post-myocardial ischemia [49,50,51]. Recent studies also pointed out the potential of targeting Wnt signaling in therapeutic applications. For instance, inhibiting the Wnt pathway has shown promise in reducing fibrosis and improving cardiac function after myocardial injury [52] (Figure 2).

Inhibition of Wnt has been shown to have beneficial effects on the infarcted myocardium [49,53,54]. Modulation of the Wnt signaling pathway provides a potential pharmacologic target for regenerative signaling in damaged myocardial tissue. Several groups of inhibitors of Wnt signaling have been investigated to alter the prognosis of myocardial fibrosis after ischemic injury (Table 1). Natural antagonists of the Wnt pathway, secreted Frizzled proteins Sfrp1, Sfrp2, and Sfrp4, have been shown to reduce fibrosis and improve cardiac function [54,55,56]. Sfrp1 has also been found to protect cardiac cells from hypoxia and reperfusion injury [57]. On the other hand, loss of Sfrp1 leads to impaired cardiac function in mice [58]. Several lines of evidence document a beneficial effect of the administration of porcupine inhibitors, which subsequently block Wnt secretion, on reducing adverse cardiac remodeling and subsequent fibrosis. These inhibitors have also demonstrated a beneficial effect on cardiomyocyte proliferation and cardiac regeneration after myocardial infarction [59,60]. Another way to inhibit canonical Wnt signaling is by stabilizing the destruction complex. Frequently used agents are tankyrase inhibitors that are involved in axin degradation. The inhibition of tankyrases, thus, facilitates axin stabilization and leads to alleviated β-catenin degradation. XAV939, a tankyrase inhibitor, was able to suppress fibrosis, promote angiogenesis, and reduce infarct size after myocardial infarction by blocking the Wnt/β-catenin signaling pathway in mice [61]. Moreover, in an isoproterenol-induced HF model in zebrafish, administration of XAV939 protected against ventricular dilatation and cardiac dysfunction by suppressing excessive activation of Wnt/β-catenin signaling [62]. Inhibiting the Wnt signaling pathway was effective at reducing fibrosis and improving cardiac function after myocardial injury [52].

Pharmacological inhibitors of Wnt signaling attenuate cardiac hypertrophy and fibrosis, thereby improving cardiac function in animal models of pressure overload-induced heart failure. ICG-001 is a small molecule that specifically blocks the interaction between β-catenin and its coactivator, the CREB-binding protein (CBP). These effects are associated with an inhibition of β-catenin–mediated gene transcription. Several studies documented that the inhibition of Wnt signaling using ICG-001 has beneficial effects on the infarcted myocardium [49,53,54]. It has been shown that administration of ICG-001 post-myocardial infarction improved contractile function in chronically infarcted rat myocardium. The inhibition of β-catenin-mediated transcription led to an improvement in the ejection fraction (EF) at 10 days post-MI [64]. The blockade of Wnt/β-catenin by ICG-001 also improved cardiac injury and restored heart function in a mouse model of cardiac hypertrophy and heart failure induced by transverse aortic constriction (TAC) [70]. Similar attenuation of cardiac hypertrophy and fibrosis in the left ventricular wall in mice heart with TAC as a consequence of the inhibition of the Wnt/β-catenin pathway through the administration of ICG001 was documented by Methatham et al. [65]. The beneficial effect of ICG-001 on cardiac function was also found in an in vivo model of chronic myocardial infarction in female rats [71].

After ischemic cardiac damage, the cellular population of the heart changes rapidly. Neutrophils, macrophages, fibroblasts, endothelial cells, and epicardial cells interact with each other and with cardiomyocytes. This is accompanied by changes in fibroblast activation, extracellular matrix deposition, and neovascularization. The process of wound healing related to ischemia in the myocardium consists of different phases, including inflammation, the formation of granulation tissue, and a maturation phase in which a scar is formed. Wnt/β-catenin signaling has been found to play a role in each of these phases [21,52,72]. Cardiomyocyte death evokes an early inflammatory response. It is triggered by the release of chemokines and cytokines from damaged cardiomyocytes and begins with the invasion of polymorphonuclear neutrophils (PMNs) into the infarct area from the 12th to 16th hour after infarction. PMNs help to remove dead cardiomyocytes. Their infiltration is followed by an influx of other inflammatory cells such as lymphocytes, plasma cells, and macrophages (from 3 days after infarction). During the inflammatory phase, necrotic debris is cleared and angiogenesis is activated [73]. Increasing evidence suggests that Wnt may modulate the inflammatory response of the body [74,75,76]. It has been documented that activation of noncanonical Wnt signaling via Wnt5a regulates the expression of pro-inflammatory genes, such as interleukin (IL)-6, IL-1b, and IL-8, and cardiomyocyte-specific overexpression of the Wnt inhibitor Wif1 has been found to play an important role in modulating the inflammatory response and improving infarct healing [74,75]. In addition, administration of the porcupine LGK-974 inhibitor was found to attenuate the inflammatory response after ischemia in the mouse heart by blocking Wnt signaling [63]. A protective effect of reducing Wnt signaling activity was also demonstrated by Palevski et al., who proved that the loss of Wnt secretion by macrophages has a beneficial effect on wound healing after myocardial infarction [77]. The influence of canonical Wnt/β-catenin signaling on the modulation of the inflammatory response has also been demonstrated. Overexpression of β-catenin leads to increased production of pro-inflammatory cytokines in cardiomyocytes [67]. The administration of Huoxine tablets, a compound used mainly in traditional Chinese medicine that has previously been reported to have a potent inhibitory effect on Wnt/β-catenin signaling [68], has a role in protecting against myocardial ischemic injury through the prevention of a detrimental Wnt/β-catenin signaling-mediated inflammatory response [78]. However, some studies show that regulation of the inflammatory response by the activation of the Wnt signaling pathway may have a beneficial effect on recovery after myocardial infarction. Triggering of the canonical Wnt signaling pathway through the inhibition of GSK3β has been shown to promote differentiation into anti-inflammatory macrophages. Moreover, overexpression of Wnt10b and Wnt11 in myocardial tissue led to a significant reduction in the inflammatory response in the infarcted heart, increased neovascularization, and improved ventricular function [79,80].

Three to five days after infarction, there is a decrease in inflammatory signals and a current upregulation of profibrotic signals, indicating the beginning of the reparative phase of wound healing after infarction. This phase is dominated by activated anti-inflammatory macrophages, activated fibroblasts, and endothelial cells [81]. Macrophages secrete the anti-inflammatory cytokine IL-10 and growth factors including transforming growth factor-β (TGF-β). Activated cardiac fibroblasts exhibit uncontrolled proliferation and overproduction of the extracellular matrix (ECM) and can differentiate into myofibroblasts that express contractile proteins, smooth muscle actin (SMA), and embryonic smooth muscle myosin, as well as secrete large amounts of matrix proteins that form collagen scars. The cytokine TGF-β plays a key role in the differentiation of fibroblasts into myofibroblasts [82]. There is increasing evidence that canonical Wnt3 signaling acts in concert with TGF-β signaling in myofibroblast differentiation [83], which may be related to an increase in IL-11 production [84]. Exosomes containing Wnt3a and Wnt5a proteins have also been found to functionally contribute to cardiac fibrosis [85]. Activation of the Wnt/β-catenin and TGF-β signaling pathways via NET1 (Neuroepithelial cell-transforming 1) promotes collagen synthesis in fibroblasts and is thus involved in the development of cardiac fibrosis in mice [86]. It has been shown that after myocardial infarction there is an increase in the expression of Wnt1, which subsequently acts through autocrine and paracrine mechanisms to induce the cardiac fibroblast activation, proliferation, and expression of pro-fibrotic genes such as collagen and endothelin. This effect of Wnt1 is dependent on the β-catenin signaling pathway and is associated with little collagen deposition and impaired cardiac function [52]. The involvement of Wnt signaling in the process of adverse ventricular remodeling and in the development of cardiac fibrosis has also been demonstrated in other studies. Transcription factor EB has been shown to inhibit the differentiation of fibroblasts into myofibroblasts, thereby reducing collagen I and III expression in mice after MI. This effect was mediated by the formation of the TFEB-β-catenin-TCF/LEF1 complex, which alters the gene expression profile of β-catenin [87]. Activation of Proline/arginine-rich and leucine-rich repeat protein (PRELP), which has been identified as an upstream regulator of the Wnt/β-catenin signaling pathway, is also associated with the overexpression of MMP-9 and TIMP-1, which are proteins responsible for regulating extracellular matrix remodeling [25]. Increased expression and secretion of Wnt2 and Wnt4 were found after MI. Elevated Wnt2 and Wnt4 activate β-catenin/NF-kB/p65 through the cooperation of the LRP6 and Fzd4/2 receptors, which accelerates MI-induced cardiac fibrosis in patients with MI [88]. The involvement of noncanonical Wnt signaling in the promotion of myocardial inflammation and fibrosis was documented by Abraityte et al., who showed that Wnt5a can stimulate fibroblasts to secrete the pro-inflammatory cytokine interleukin-6 (IL-6) and tissue inhibitor of metalloproteinase-1 (TIMP-1) [89]. Furthermore, silencing of the Wnt5a gene leads to the abrogation of cardiac fibroblast differentiation induced by the overexpression of Wnt5a and the transcription factor Prrx2 (paired-related homeobox 2) via the effect of TGF-β in cultured cardiac fibroblasts [90]. Triggering the canonical Wnt/β-catenin signaling using recombinant Wnt3a can induce fibroblast differentiation [66]. Conversely, attenuation of this pathway had a protective effect on the progression of cardiac fibrosis and was associated with a reduction in the expression of β-catenin, phosphorylated GSK-3β, Wnt3a, and Wnt1 [69,91]. These findings suggest that the administration of Wnt inhibitors could play a protective role in wound healing after MI. In a model of myocardial infarction, inhibition of Wnt signaling has been shown to reduce collagen concentration and improve cardiac function [56], while the administration of the natural Wnt/β-catenin inhibitors, Liensin and Linggui Zhugan, delayed cardiac hypertrophy and improved cardiac function [69]. The inhibition of β-catenin signaling also ameliorates angiotensin II-induced myocardial fibrosis and restores cardiac function, as well as lowers blood pressure [66]. 

The blood supply to the myocardium damaged by ischemia must be restored by the formation of new blood vessels, and a scar must be created to harden the damaged area. At the cellular level, these processes include proliferation, migration, and differentiation, with a specific role for a large number of growth factors (e.g., vascular endothelial growth factor-VEGF) and angiogenic molecules. Although the inhibition of Wnt signaling after MI by Pyrvinium was shown to have a positive effect on promoting angiogenesis through the inhibition of adverse cardiac remodeling [44], and another Wnt pathway inhibitor, Dkk2, has also been found to activate LRP6 and stimulate angiogenesis in endothelial cells [40], numerous studies have documented that the positive effect on angiogenesis is due to the activation of Wnt signaling [92,93]. Endothelial cell invasion was shown to be regulated by lymphoid enhancer-binding factor 1 (Lef1), a target gene of the Wnt/β-catenin pathway, which was associated with the increased expression of matrix metalloproteinase 2 [94]. Wnt5a expression in endothelial cells promotes angiogenesis. Its increased expression has been linked to the upregulation of several other regulators of angiogenesis, such as matrix metalloproteinase-1, interstitial collagenase, and Tie-2, a receptor for angiopoietins [95]. Activation of the noncanonical Wnt11 pathway has been reported to promote angiogenesis and improve cardiac function [96]. Furthermore, Wnt1 was found to have an effect on the regulation of angiogenesis and a positive correlation was detected between higher levels of Wnt1 expression and VEGF in a rat atherosclerotic model [97]. 

## 6. Conclusions

In summary, several studies suggest that Wnt signaling plays an important role in the regulation of cellular homeostasis and that dysregulation of this signaling represents an important factor involved in the development of pathological changes in the cardiovascular system. Wnt pathways also play an important role in the regulation of changes in heart function after myocardial infarction. In early phases of responses, Wnt signaling activation, particularly Wnt/β-catenin signaling, can serve as a potential promoter of the healing and repairing of the heart after myocardial infarction. However, in later phases this activation is associated with negative pathological heart tissue remodeling, myocardial fibrosis development, and apoptosis, and thus is involved in heart disease progression. 

The current information indicates that the modulation of the Wnt signaling pathway provides a potential pharmacologic target for regenerative signaling in damaged myocardial tissue. Several groups of Wnt signaling inhibitors have been investigated for altering the prognosis of myocardial fibrosis and pathological tissue remodeling after myocardial ischemic injury. Their protective effects can play an important role in the inhibition of the Wnt/β-catenin-induced modulation of matrix metalloproteinases, which are enzymes that play a crucial role in extracellular matrix remodeling. Searching for Wnt signaling inhibitors that influence the function of gap junction protein connexin 43 through β-catenin inhibition can be promising for restoring cardiac function through the elimination of the occurrence of life-threatening arrhythmias. 

We have summarized the mass of evidence that demonstrates the protective effect of Wnt signaling inhibitors in attenuating the consequences of ischemic injury. However, some questions need to be answered in this context, such as how the crosstalk between Wnt signaling and other signaling pathways may modulate the downstream activities of Wnt signaling and may further influence outcomes. In addition, many key components of Wnt signaling also play important roles in Wnt-independent molecular pathways, and their inhibition may affect their roles within them. Therefore, a better understanding of the precise mechanisms of cardiovascular disease, the involvement of Wnt signaling in its occurrence, and the recognition of the common role of Wnt signaling inhibitors in cardioprotection mechanisms is important for the better management of patients with ischemia. Moreover, there is still a need to develop and optimize inhibitors with minimal toxicity and high selectivity without affecting other molecular pathways. This will provide an opportunity to apply the results of basic studies into clinical trials, ultimately enabling the evaluation of Wnt-dependent therapeutic intervention in the human cardiovascular system.

## Figures and Tables

**Table 1:** Wnt signaling pathway inhibitors in ischemic cardiac diseases.

Group of Inhibitors	Model	Sample	Effect	References
Natural Wnt antagonists				
Sfrp1	MI (myocardial infarction)	transgenic mice overexpressing Sfrp1	Improved inflammatory response.	[55]
	In vitro model of ischemia/reperfusion	H9c2 cells	Directly protected cells from hypoxia and reperfusion injury and reoxygenation-induced apoptosis through inhibition of the Wnt signaling pathway.	[57]
	Sfrp1 knock-out	aged sFRP-1 KO mice	Loss of Sfrp1 led to impaired cardiac function and massive cardiac fibrosis.	[58]
Dkk1 and Dkk2	In vitro experiments; MI	HUVECs, rats, mice	Dkk2 promoted angiogenesis in cultured human endothelial cells and in in vivo mouse assays. Dkk1, in contrast, suppressed angiogenesis. Local injection of Dkk2 protein significantly improved tissue recovery with increased neovascularization in animal models.	[40]
Porcupine inhibitors				
WNT-974 (PORC) 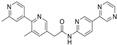	MI	C57BL/6 mice	WNT-974 improved recovery of cardiac function after myocardial infarction. Damaged heart tissue exposed to WNT-974 showed reduced scarring and reduced production of collagen 6.	[59]
LGK-974 (PORC) 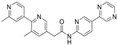	In vitro experiments; ischemia/reperfusion injury	RAW 264.7 macrophages, HL-1 cardiomyocytes, mice	Animals treated with LGK-974 showed an attenuated inflammatory response and improved cardiac function. In an in vitro model, LGK974 inhibited the activation of Wnt signaling in monocytes/macrophages and reduced their pro-inflammatory phenotype.	[63]
CGX1321 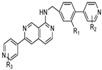	MI	mice	CGX1321 improved cardiac function, and reduced myocardial infarct size and cardiac fibrosis after MI.	[60]
CREB-binding protein (CBP) inhibitors				
ICG-001 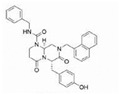	MI	female Sprague Dawley rats	ICG-001 improves cardiac contractile function in a myocardial infarction model.	[64]
	TAC		ICG001 prevents cardiac hypertrophy and fibrosis by regulating immune activation and the Wnt/β-catenin signaling pathway and by inhibiting the inflammatory response involving macrophages.	[65]
	Chronic infusion of angiotensin II	rats	ICG-001 ameliorated myocardial fibrosis and inhibited the expression of α-smooth muscle actin, fibronectin, and collagen I.	[66]
Tankyrase inhibitors				
Pyrvinium 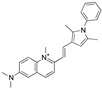	MI	mice	Pyrvinium reduced adverse cardiac remodeling as demonstrated by a decrease in the left ventricular internal diameter in diastole.	[44]
XAV939 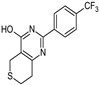	Isoproterenol-induced HF	zebrafish	Inhibition of TNKS activity protected against ventricular dilatation and cardiac dysfunction and abrogated overactivation of Wnt/β-catenin signaling.	[62]
Other Wnt signaling inhibitors				
UM206	MI	male Swiss mice	Blocking of Frizzled signaling reduces infarct expansion and preserves cardiac function after MI.	[54]
Huoxin formula	MI	mice	HXP administration reliably protected against heart damage induced by infarction, reduced infarct size, and improved cardiac function. HXP protected against oxidative stress-induced damage and suppressed the inflammatory response induced by MI.	[67,68]
Linggui Zhugan decoction formula	Heart failure	rats	Linggui Zhugan decoction improved cardiac function and reduced the deposition of collagen fiber.	[69]

## Data Availability

Not applicable. No data were generated or analyzed in this work.
